# Advances in the detection of antimicrobial resistance in aquatic environments: a methodological perspective

**DOI:** 10.1093/biomethods/bpag029

**Published:** 2026-06-04

**Authors:** Olukayode Adebola Ibitoye, Chinyere Nkemjika Anyanwu, Abdulganiy Babatunde Agbaje, Ilemobayo Victor Fasogbon, Reuben Samson Dangana, Saheed Adekunle Akinola, Julius Tibyangye, Adam Ahmed Adam, Patrick Maduabuchi Aja

**Affiliations:** Department of Microbiology and Immunology, Kampala International University—Western Campus, Bushenyi, Uganda; Department of Microbiology and Immunology, Kampala International University—Western Campus, Bushenyi, Uganda; Department of Microbiology and Parasitology, School of Medicine and Pharmacy, College of Medicine and Health Sciences, University of Rwanda, Huye, Rwanda; Department of Biochemistry, Kampala International University—Western Campus, Bushenyi, Uganda; Discipline of Genetics, School of Life Sciences, University of Kwazulu-Natal, Westville, Durban, 3629, South Africa; Department of Microbiology and Parasitology, School of Medicine and Pharmacy, College of Medicine and Health Sciences, University of Rwanda, Huye, Rwanda; Department of Microbiology and Immunology, Kampala International University—Western Campus, Bushenyi, Uganda; Department of Microbiology and Immunology, Kampala International University—Western Campus, Bushenyi, Uganda; Department of Biochemistry, Kampala International University—Western Campus, Bushenyi, Uganda

**Keywords:** antimicrobial resistance (AMR), resistome profiling, artificial intelligence (AI), machine learning (ML), metagenomics, environmental microbiome

## Abstract

Antimicrobial resistance (AMR) is a global health and environmental challenge, driven by complex interactions among microbial communities, resistance genes, and selective pressures in various ecological niches. Traditional surveillance procedures often fall short in capturing the full diversity and dynamics of resistance reservoirs in the environment. This review examines the integration of artificial intelligence (AI) and machine learning (ML) with next-generation sequencing (NGS) technologies for comprehensive resistome profiling. We discuss advances in multi-omics approaches, particularly metagenomics, microbiome-based analytics, and metatranscriptomics. We also highlight computational workflows that enable high-resolution mapping of resistance genes, their mobile genetic elements, and host associations. The role of AI/ML in resistome prediction, classification, and source tracking, as well as the incorporation of environmental metadata for contextual interpretation is discussed based on the selected literature. Moreover, we assess current challenges and propose future directions for developing standardized, scalable, and interpretable bioinformatic pipelines in AMR surveillance. This review primarily elucidates the potential of integrated AI-omics platforms to revolutionize aquatic environmental AMR monitoring and inform risk assessment and mitigation strategies.

## Introduction

Antimicrobial resistance (AMR) stands as one of the most pressing global public health threats of the 21st century [1]. The ever-increasing incidence of infections caused by antibiotic-resistant microorganisms jeopardizes the effectiveness of conventional antibiotic therapies, resulting in increased morbidity, mortality, and healthcare costs worldwide [2]. While clinical settings have traditionally been the primary focus of AMR surveillance, it is increasingly recognized that environmental reservoirs, particularly aquatic ecosystems, play a critical role in the emergence, persistence, and distribution of antimicrobial resistance genes (ARGs) and antibiotic-resistant bacteria (ARB) [3].

Aquatic environments serve as significant nexus points where antibiotics, heavy metals, disinfectants, and resistant microorganisms converge from various anthropogenic sources, such as agricultural runoff, industrial effluents, and human sewage [4]. This constant influx creates selective pressures that favour the proliferation and horizontal gene transfer (HGT) of ARGs among diverse microbial communities [5]. Therefore, the environment acts as an incubator for the development of new resistance mechanisms and also as a source that spreads the resistance back to human and animal populations. This highlights the interconnection of environmental, animal, and human health, a concept central to the One-Health approach [6].

This review synthesizes recent methodological advances in aquatic environmental antimicrobial resistance detection; it does not present new experimental data. It aims to provide an overview of the current molecular tools employed in the surveillance of aquatic antibiotic resistance. Furthermore, it explores cutting-edge innovations and prospects for rapid and comprehensive resistome profiling, such as clustered regularly interspaced short palindromic repeats (CRISPR)-based diagnostics, nanopore sequencing, and multi-omics integration. It also addresses critical gaps, limitations, and the urgent need for standardized protocols in environmental AMR surveillance, alongside discussing the policy implications and practical applications of these advanced molecular tools in safeguarding public and ecological health. By synthesizing current knowledge and highlighting future directions, this review seeks to contribute to a more robust and proactive global response to the escalating challenges of AMR.

## Literature selection

The literature included in this review was identified through searches of Web of Science, Scopus, and PubMed databases. Keywords included combinations of ‘environmental antimicrobial resistance’, aquatic resistome’, ’metagenomics’, ‘AMR detection’, and ‘surveillance methods’.

### Inclusion and exclusion criteria

Articles published between 2010 and 2025 were considered. Studies were selected based on relevance to methodological development or application in environmental monitoring, while non-peer-reviewed sources and unrelated clinical-only studies were excluded.

## Culture-based and culture-independent techniques for AMR surveillance

The study of microbial communities and ARGs in aquatic environments has traditionally relied on culture-based methods. These foundational techniques provide direct phenotypic evidence of resistance by isolating bacteria on selective media under laboratory conditions [7]. These methods allow for the phenotypic characterization of specific strains, including testing for antibiotic susceptibility and resistance profiles [8]. Culture-based approaches remain essential for validating functional resistance traits, isolating viable pathogens, and performing downstream analyses such as whole-genome sequencing or conjugation assays. They are especially useful in public health and clinical microbiology, where identifying culturable, pathogenic strains remains a priority [7].

However, the utility of culture-dependent techniques is fundamentally constrained by a major limitation in which a substantial proportion of the environmental microorganisms may be unculturable under standard laboratory conditions [9]. Many environmental bacteria exist in dormant or low-nutrient-adapted states, require complex interspecies interactions, or depend on specific environmental conditions that are difficult to replicate in vitro [10]. As a result, culture-based methods grossly underestimate microbial diversity and overlook many important taxa involved in ecological processes or resistance gene exchange in natural systems [11]. Thus, their analytical sensitivity and environmental representativeness are limited by cultivation biases. Environmental studies indicate that culture-dependent approaches may recover only a fraction of microbial diversity present in environmental samples, particularly when organisms require specialized growth conditions or exist in viable but non-culturable states (VBNC) [10, 12].

Consequently, rare or slow-growing resistant organisms may remain undetected. From a sensitivity perspective, culture-based detection typically requires 10^1^–10^2^ colony forming units (CFU) per milliliter to reliably detect bacteria in environmental samples, depending on the medium and incubation conditions [13]. While culture methods allow direct confirmation of viable organisms and enable phenotypic antibiotic susceptibility testing, their applicability in environmental surveillance is limited by low recovery rates and bias towards fast growing taxa [10] (Table 2).

**Figure 1:**
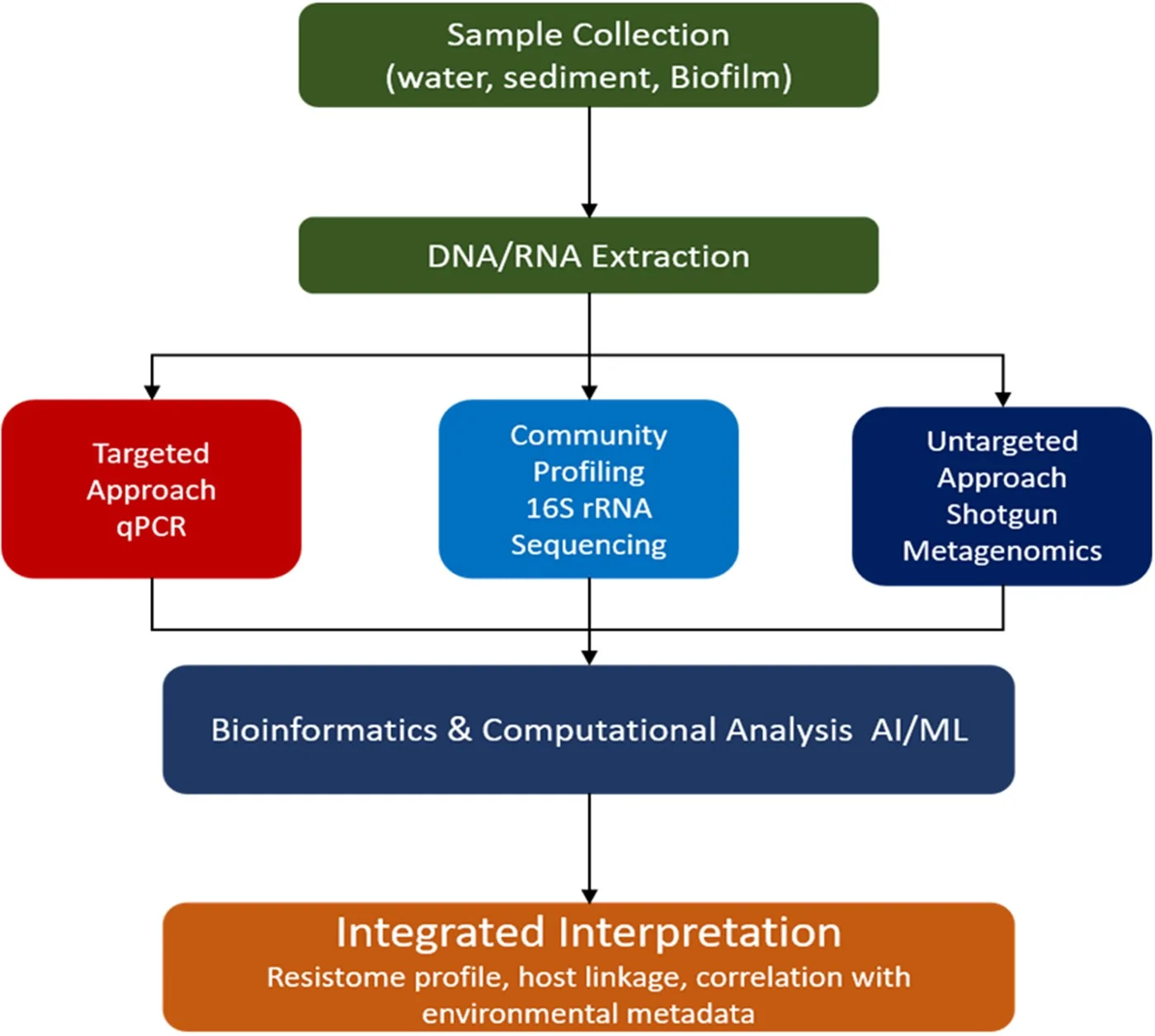
Schematic workflow for culture-independent molecular surveillance of aquatic resistomes.

To address these limitations, culture-independent methods have become the basis of modern microbial ecology and resistome surveillance. These techniques do not require cultivation and instead analyse deoxyribonucleic Acid (DNA) or ribonucleic Acid (RNA) extracted directly from environmental samples, allowing researchers to profile microbial communities and detect ARGs with much greater resolution and sensitivity [9]. High-throughput molecular techniques such as 16S rRNA gene sequencing, quantitative polymerase chain reaction (qPCR), and shotgun metagenomics have revolutionized our understanding of microbial diversity and the functional of resistomes in aquatic ecosystems [14]. Figure 1 illustrates an overview of the culture-independent molecular workflow for environmental microbial and resistome analysis, beginning with the collection of samples through series of molecular techniques, then bioinformatics and computational analysis pipeline, often supported by artificial intelligence or machine learning tools. The final step involves integrated interpretation to generate resistome profiles, link resistance genes to potential hosts, and correlate findings with environmental metadata [15].

Despite these limitations, culture-based approaches remain important for confirming the phenotypic expression of antimicrobial resistance detected through molecular assays and for associating resistance genes with specific bacterial isolates [16].

The process begins with field sample collection, followed by DNA/RNA extraction, high-throughput sequencing, bioinformatics analysis (often incorporating AI/ML tools), and concluding with integrated interpretation to link resistance genes to environmental metadata.

Culture-independent methods provide several distinct advantages; they enable the detection of both culturable and non-culturable organisms, capturing the full spectrum of microbial diversity [17]. They facilitate the comprehensive profiling of resistomes, including known and novel ARGs, and can identify mobile genetic elements (MGEs) like plasmids and integrons that mediate HGT [5], and these tools allow researchers to link microbial community shifts with environmental gradients, such as pollution intensity or seasonal variation. Despite their strengths, culture-independent methods also present challenges. Reference [18] reported that they often require sophisticated bioinformatics tools, specialized sequencing platforms, and access to well-curated databases for gene annotation. Interpretation of metagenomic data can be complex, especially when differentiating between active and dormant genes or when contamination and sequencing artefacts lead to false positives. Nevertheless, the depth of insight these methods offer far surpasses traditional techniques, particularly in environments such as rivers, wastewater systems, and sediments, where microbial communities are dense, diverse, and frequently exposed to anthropogenic stressors [19].

### 16S rRNA gene sequencing in microbial community profiling

One of the most widely used molecular tools for profiling microbial communities in aquatic environments is 16S ribosomal ribonucleic Acid Gene (16S rRNA) gene sequencing. This technique targets a specific region of the 16S rRNA gene, which encodes the small subunit of the prokaryotic ribosome which is present in all bacteria and archaea. The gene contains conserved sequences interspersed with hypervariable regions that are unique to different taxonomic groups, making it an ideal genetic marker for identifying and classifying microbes [20]. In environmental surveillance, especially in freshwater and wastewater ecosystems, 16S rRNA gene sequencing has been the standard approach for taxonomic profiling [21]. It enables researchers to detect shifts in microbial community composition, monitor biodiversity, and explore ecological interactions under the influence of pollutants, seasonality, or land use changes. This is particularly valuable in systems affected by industrial effluents, where microbial communities serve as sensitive bioindicators of environmental health and anthropogenic stress [22].

The method typically involves extracting total DNA from environmental samples followed by PCR amplification of selected hypervariable regions (commonly V3–V4 or V4–V5) of the 16S rRNA gene [23]. These amplified regions are then sequenced, providing massive amounts of sequence data that can be analyzed using bioinformatics pipelines to determine the composition and relative abundance of microbial taxa [24]. One of the main advantages of 16S rRNA gene sequencing is its cost-effectiveness and scalability. Compared to more comprehensive approaches like shotgun metagenomics, 16S rRNA gene sequencing requires less computational power and is more accessible for routine surveillance, especially in resource-limited settings [25]. It also enables broad ecological insights by identifying dominant phyla, genera, or families and comparing microbial diversity across spatial or temporal gradients [25]. In the context of AMR surveillance, 16S rRNA gene sequencing data can be used to identify potential hosts of ARGs, track ecological disturbances in riverine systems, and guide targeted studies using more specific resistome tools [26]. Reference [27] reported that an increase in Proteobacteria abundance downstream from industrial discharge points may suggest an elevated ARGs risk, warranting further investigation through qPCR or metagenomics. While 16S rRNA gene sequencing is the gold standard for taxonomic profiling in microbial ecology, it has significant limitations when it comes to detecting functional genes, like ARGs, MGEs, and metabolic pathways or enzymatic functions. These functional attributes require shotgun metagenomic sequencing, metatranscriptomics, or functional annotation tools beyond the 16S region [25]. Additionally, the resolution of this method is often limited to the genus level, and closely related species may remain indistinguishable due to high sequence similarity in the 16S rRNA gene region [28]. Another challenge lies in the potential for amplification bias, where some taxa may be over- or underrepresented depending on primer choice and PCR conditions [29]. Despite these limitations, 16S rRNA gene sequencing remains a cornerstone, particularly as a complementary method to more advanced functional genomics approaches [30].

### Shotgun metagenomics for functional and taxonomic resolution

Among one of the most powerful tools available for comprehensive microbial and resistome analysis in environmental samples is shotgun metagenomic sequencing [31, 32]. Unlike targeted approaches such as 16S rRNA gene sequencing, which amplify and sequence a specific gene, shotgun metagenomics involves the unbiased sequencing of all DNA present in a sample [33]. This method provides both taxonomic and functional insights, offering a high-resolution view of microbial communities and their associated genes, including ARGs, MGEs, virulence factors, and metabolic pathways [34]. Comparative benchmarking studies have shown that metagenomic sequencing may fail to detect low-abundance ARGs that are readily identified by qPCR, particularly when sequencing depth is limited [35, 36]. In wastewater surveillance, qPCR detected ARGs in treated effluent that metagenomics could not identify due to insufficient read coverage [36]. However, sequencing-based methods have a higher practical detection threshold. ARGs typically must represent approximately 10^−5^ to 10^−6^ of the total metagenomic reads to be reliably detected, depending on sequencing depth and database coverage [35] (Table 2).

In aquatic ecosystems microbial communities are often exposed to a complex mixture of pollutants, including antibiotics, heavy metals, and organic contaminants [37]. These stressors drive microbial adaptation and resistance evolution, necessitating a surveillance tool that can simultaneously detect both the organisms present and their functional capabilities [38]. Shotgun metagenomics fulfils this role by enabling the detection, identification, and quantification of known and novel ARGs, even in unculturable bacteria [31]. The standard workflow begins with total DNA extraction from the environmental matrix, followed by random fragmentation of the DNA and sequencing [14]. The resulting reads are then assembled and annotated using bioinformatics pipelines, where they are compared against curated databases such as the Comprehensive Antibiotic Resistance Database (CARD), ResFinder, or ARG-ANNOT to identify resistance genes and their variants [39]. One of the key advantages of shotgun metagenomics is its ability to capture the full genetic repertoire of the microbial community, including rare or previously unknown genes [40]. It also allows researchers to explore the co-occurrence of ARGs with MGEs, such as plasmids, integrons, and transposons, which are instrumental in facilitating HGT, a major mechanism driving the spread of AMR in aquatic environments [41]. In addition, this approach can reconstruct the metabolic and ecological roles of microbial taxa, helping to elucidate how pollution affects nutrient cycling, detoxification, and ecosystem resilience [42]. Despite its immense potential, shotgun metagenomics is resource-intensive. It requires significant computational infrastructure and expertise in bioinformatics to process, assemble, and interpret large volumes of sequence data [39].

Additionally, the cost of sequencing limits its application in routine monitoring, especially in low-income regions. Another challenge lies in the accurate annotation of genes, as misannotations and false positives may occur due to sequence homology between ARGs and non-resistance genes or the presence of pseudogenes [43]. Nevertheless, the increasing availability of cloud-based analysis platforms and machine learning–based classifiers (such as DeepARG) is rapidly improving the scalability and precision of metagenomic ARG detection [44].

Moreover, combining metagenomic data with metadata on environmental variables like pH, conductivity, pollutant concentrations enhance our ability to model and predict resistome dynamics under varying ecological pressures [45]. Shotgun metagenomics has been instrumental in studies revealing the widespread dissemination of clinically relevant ARGs, such as blaNDM-1, mcr-1, and sul1, in rivers receiving untreated industrial or hospital effluents [46]. These discoveries highlight the importance of implementing metagenomic surveillance in AMR risk assessment and policy development.

### qPCR for resistome profiling

Profiling the resistome, which is the full complement of ARGs in a microbial community, requires tools that can detect and quantify both known and emerging resistance determinants with precision [47]. A variety of molecular techniques have been developed and refined for this purpose, ranging from targeted approaches like qPCR to broad-spectrum, computationally intensive strategies such as metagenomic sequencing coupled with machine learning algorithms [48]. One of the most widely used tools for resistome surveillance is qPCR. qPCR assays typically achieve detection limits of 1–10 gene copies per reaction, enabling detection of ARGs even in highly diluted environmental samples [49–51]. This high analytical sensitivity makes qPCR particularly effective for monitoring low-abundance resistance genes (Table 2). This method amplifies specific DNA sequences using fluorescent dyes or probes, allowing for the real-time quantification of selected ARGs. It is especially useful for detecting high-priority resistance genes, such as blaNDM-1, mcr-1, sul1, and tetA, in environmental samples. qPCR provides robust quantification across a dynamic range of 10^1^–10^8^ gene copies, but accuracy depends heavily on primer design, amplification efficiency, and normalization strategies [52] (Table 2). The technique is highly sensitive, cost-effective, and relatively fast, making it ideal for routine monitoring, even in resource-limited settings [53]. However, qPCR is inherently targeted, meaning it requires prior knowledge of the gene sequences of interest and is limited by primer specificity. Because primers must be designed for known gene sequences, qPCR cannot detect novel or unexpected resistance genes. Additionally, primer mismatches can produce false negatives or underestimation of gene abundance [49]. This can result in missed detection of novel or mutated ARGs and restricts the scope of analysis to a predefined panel of genes [54]. To expand beyond the limitations of traditional qPCR, researchers have turned to high-throughput quantitative polymerase chain reaction (HT-qPCR) platforms, such as the Wafergen SmartChip or Fluidigm BioMark systems [55]. These technologies enable simultaneous amplification of hundreds of ARG targets, improving gene coverage and detection sensitivity [56]. While powerful, HT-qPCR systems require specialized instrumentation and carry higher operational costs, which can be a barrier to widespread adoption [50]. Comparative studies evaluating wastewater resistomes demonstrate that qPCR can detect ARGs that are below the detection threshold of metagenomic sequencing, especially in samples with low microbial biomass [57]. Reference [36] reported that a comparative analysis of wastewater samples showed that qPCR detected several ARGs in treated effluent that were not identifiable through metagenomic sequencing due to insufficient sequencing depth. Consequently, qPCR is most appropriate for targeted monitoring programs, such as tracking clinically relevant genes like blaCTX-M, tetA, or sul1 in wastewater treatment systems [58] (Table 2).

In contrast to targeted methods, shotgun metagenomic sequencing, as discussed previously, enables unbiased, community-wide resistome analysis. When paired with curated ARG databases, metagenomic data can be used to identify, classify, and track both known and potentially novel ARGs [59]. These platforms employ homology-based alignment tools, which compare DNA or amino acid sequences from environmental reads to reference sequences in ARG repositories [60]. Recent innovations have introduced machine learning algorithms into resistome profiling, most notably through tools such as DeepARG [44]. DeepARG uses deep neural networks trained on extensive datasets to predict ARGs with high accuracy, even when the sequence similarity to known genes is low. This makes it especially valuable for detecting novel or cryptic resistance genes that traditional alignment-based methods might overlook. In addition to enhancing detection capabilities, AI-based tools can also reduce the burden of false positives, improve gene annotation consistency, and accelerate large-scale resistome analysis [44]. Another category of tools includes bioinformatics pipelines such as ARGs-OAP, ResFinder, AMRFinderPlus, and MetaStorm, which automate the steps of ARG annotation, quantification, and visualization [60]. These pipelines typically incorporate modules for quality control, host-taxonomy association, and visualization dashboards. However, effective use of these platforms requires careful quality control, particularly in distinguishing true ARGs from contaminants or horizontally transferred sequences, which may confound downstream interpretations [59]. Ultimately, the choice of tool or combination of tools for AMR Surveillance and resistome profiling (Table 1) depends on the study’s objectives, available resources, and the desired resolution of results. Targeted qPCR is optimal for quick screening and longitudinal surveillance of priority ARGs, while metagenomics and AI-enhanced platforms are better suited for exploration, large-scale studies aiming to map resistome diversity and uncover novel resistance mechanisms.

**Table 1:** Comparison of key molecular techniques for aquatic AMR surveillance.

Technique	Target	Application	Advantages	Limitations	References
**qPCR**	Pre-defined ARGs	Targeted detection and quantification	Sensitive, rapid, cost-effective, suitable for low-resource settings	Limited to known genes; cannot detect novel ARGs	[49, 53]
**16S rRNA sequencing**	16S gene regions	Microbial community profiling	Cost-effective, scalable, low computational demand	No functional/ARG data; low taxonomic resolution; bias-prone	[20, 28, 29]
**Shotgun metagenomics**	Total sample DNA	Comprehensive ARG and functional profiling	High taxonomic & functional resolution; detects novel genes	Expensive; computationally intensive; annotation challenges	[33, 39, 40]
**AI/ML Tools (e.g. DeepARG)**	Metagenomic data	ARG prediction and classification	Detects novel ARGs; improves annotation accuracy	Requires high-quality training data; potential bias	[44]

A comparative evaluation of the applicability of the AMR detection methods shows substantial variability in analytical sensitivity, quantitative accuracy, and practical applicability across the different methods. Also, the summaries of key detection thresholds and coverage which deeply elucidates important trade-off that influences method selection in aquatic environment surveillance (Table 2).

**Table 2:** Comparative summary of environmental AMR detection methods.

Method	Threshold	Coverage	Key strength	Key limitation	References
**Culture-based**	Low–moderate (∼10¹–10² CFU)	Culturable fraction only	Phenotypic validation and isolate recovery	Strong cultivation bias	[10]
**qPCR**	Very high (1–10 gene copies)	Targeted (known ARGs)	Highly sensitive and quantitative	Limited to known targets; primer bias	[52]
**Metagenomics**	Moderate (∼10^−5^–10^−6^ relative abundance)	Comprehensive resistome	Detects known and novel ARGs	Lower sensitivity for rare genes; high cost	[39]
**Nanopore sequencing**	Moderate	Comprehensive (long-read)	Resolves ARG–host linkage	Lower per-base accuracy	[66]
**CRISPR diagnostics**	Very high (∼10¹–10² copies; attomolar)	Targeted	Rapid, ultra-sensitive detection	Limited environmental validation	[62]
**AI/multi-omics**	Variable	Integrative	Predictive ARG detection and pattern analysis	Data quality and model dependence	[44]

## Emerging innovations and future prospects

### CRISPR-based diagnostics and biosensors

Emerging as a highly promising innovation for rapid and portable detection of ARGs are CRISPR-based diagnostics and biosensors [61]. Platforms such as SHERLOCK (Specific High-sensitivity Enzymatic Reporter UnLOCKing) and DETECTR (DNA Endonuclease-Targeted CRISPR Trans Reporter) leverage the precise RNA-guided DNA cleavage activity of CRISPR-Cas enzymes (e.g. Cas12, Cas13) for highly specific and sensitive nucleic acid detection [61]. These systems combine CRISPR-associated nucleases with isothermal amplification methods, enabling detection of ARGs in under 1 hour. Experimental studies have demonstrated limits of detection as low as attomolar concentrations for specific nucleic acid targets [62]. However, performance strongly depends on guide RNA design, and off-target cleavage can occur if sequence similarity exists between ARG variants. These systems typically involve a guide RNA designed to target a specific ARG sequence. Upon binding to the target, the Cas enzyme is activated, leading to collateral cleavage of reporter molecules (e.g. fluorescent or colourimetric probes), which generates a detectable signal [63]. A key advantage of CRISPR-based diagnostics is their potential for point-of-use (PoU) detection and portability, making them suitable for field-based ARG surveillance, particularly in low-resource settings [64]. They offer a rapid alternative to laboratory-bound methods, potentially enabling on-site identification of priority ARGs in aquatic environments like rivers or wastewater treatment plants [65]. This real-time capability can significantly reduce turnaround times for results, facilitating quicker decision-making for public health interventions. While still largely in the research and development phase for environmental applications, their high specificity, sensitivity, and adaptability to various sample types make them a compelling future tool for molecular AMR surveillance [65]. While promising, CRISPR diagnostics are currently best suited for targeted screening of priority resistance genes rather than comprehensive resistome profiling.

### Nanopore sequencing and real-time resistome profiling

Another groundbreaking innovation transforming resistome profiling is Nanopore sequencing, pioneered by Oxford Nanopore Technologies. This technology offers real-time, long-read sequencing by measuring changes in electrical current as DNA or RNA molecules pass through a nanoscale pore [66]. Unlike traditional sequencing platforms that require extensive sample preparation and batch processing, Nanopore sequencers are compact, relatively inexpensive, and can provide data streams as sequencing occurs. This ‘real-time’ aspect allows for immediate data analysis and rapid identification of ARGs, making it particularly advantageous for urgent public health investigations or in dynamic environmental settings [67]. The long reads generated by Nanopore sequencing are especially valuable for resistome analysis. Long reads (often exceeding 10–100 kilob) enable direct linkage between resistance genes and their bacterial hosts, which is difficult with short-read technologies [68]. They can span entire ARGs, mobile genetic elements (MGEs) like plasmids, and even resolve complex genomic rearrangements that are difficult to characterize with short-read technologies [67, 69]. This capability allows for more accurate linking of ARGs to their microbial hosts and their associated MGEs, providing crucial insights into the mechanisms of HGT and dissemination [41]. For aquatic AMR surveillance, Nanopore sequencing’s portability and ease of use present significant advantages in low-resource settings or remote field locations where access to centralized laboratories is limited. While challenges remain concerning raw read error rates and data analysis pipelines, continuous improvements in chemistry and bioinformatics are rapidly enhancing its utility for rapid and comprehensive resistome profiling [70, 71]. However, nanopore sequencing historically exhibits lower per-base accuracy (90%–95%) compared with Illumina sequencing (>99%), which can affect ARG annotation accuracy and increase false positives in some datasets. Despite this limitation, improvements in base calling algorithms and hybrid sequencing strategies have significantly improved accuracy in recent studies. As a result, nanopore sequencing is particularly valuable for mobile genetic element analysis and rapid outbreak investigations rather than routine quantitative ARG monitoring.

### Multi-omics integration

While metagenomics provides a comprehensive snapshot of the genetic potential of a microbial community, a more holistic understanding of ARG expression and functionality can be achieved through multi-omics integration [72]. This advanced approach combines data from various 'omics’ disciplines, such as metagenomics (the study of all DNA), metatranscriptomics (all RNA, indicating gene expression), and metaproteomics (all proteins, indicating active gene products) [73]. By integrating these layers of information, researchers can move beyond simply identifying the presence of ARGs to understanding which genes are actively being transcribed and translated, and thus actively contributing to resistance phenotypes in each environment [74]. Metagenomics might detect an ARG, but metatranscriptomics can reveal if that gene is currently expressed under specific environmental conditions, and metaproteomics can confirm the production of the resistance protein [75]. This level of insight is crucial for differentiating between dormant or non-functional ARGs and those that pose an immediate risk [76]. Similarly, integration of metatranscriptomics with metagenomics enables differentiation between gene presence and activity, providing more actionable surveillance insights [76]. These approaches illustrate how computational integration enhances interpretability beyond single-omics workflows. In aquatic environments, multi-omics approaches can elucidate how environmental stressors (e.g. pollutants, temperature fluctuations) influence ARG expression patterns and the overall functional resistome [77]. The computational challenges associated with integrating and interpreting such vast and diverse datasets are significant, but advancements in bioinformatics and machine learning are making multi-omics a powerful tool for a deeper, more dynamic understanding of AMR in complex ecosystems [78].

### Artificial intelligence and machine learning

The exponential growth of sequencing data in environmental AMR surveillance has necessitated the development of advanced computational tools, with artificial intelligence (AI) and machine learning (ML) emerging as transformative forces in resistome profiling [79]. These technologies move beyond traditional sequence alignment-based methods, offering enhanced capabilities for identifying, classifying, and predicting ARGs with greater accuracy and efficiency [80]. Machine learning models trained on metagenomic abundance profiles have been shown to predict ARG occurrence patterns and identify previously unrecognized resistance determinants [81]. One prominent example is DeepARG, which utilizes deep neural networks trained on extensive datasets of known ARGs. Unlike homology-based tools that rely solely on sequence similarity, DeepARG can predict ARGs with high accuracy even when the sequence identity to known genes is low [82]. This capability is particularly valuable for detecting novel or cryptic resistance genes that conventional methods might overlook, thereby accelerating the discovery of new resistance mechanisms in diverse aquatic environments [83]. Beyond gene identification, ML and AI are playing an increasingly critical role in AMR surveillance, especially in identifying and managing ARGs in the environment. One of the most impactful applications is the prediction of ARG hotspots. These applications demonstrate that machine learning classifiers trained on metagenomic abundance matrices can improve prediction of antimicrobial resistance determinants in wastewater surveillance programs [81]. By integrating extensive environmental metadata, such as physicochemical parameters, pollutant concentrations, and land-use information, with resistome profiles, machine learning models can reveal high-risk areas where ARGs are likely to accumulate and spread. These include regions in rivers, wastewater treatment systems, and agricultural runoff zones. AI excels at detecting complex, non-linear patterns that often elude traditional analytical methods, enabling more precise, targeted intervention strategies [84].

Beyond mapping ARG hotspots, AI is increasingly used to uncover novel resistance mechanisms. Unsupervised learning algorithms can analyse large-scale metagenomic datasets to identify previously uncharacterized genetic elements or resistance pathways. These tools can detect subtle anomalies in sequence data, flagging potential new ARGs for further laboratory validation [44, 82].

Moreover, AI enhances the accuracy of ARG annotation by reducing false positives that frequently arise from sequence similarities between ARGs and non-resistance genes. This not only improves the reliability of ARG databases but also ensures higher confidence in downstream risk assessments. In addition, AI-driven predictive models are being developed to simulate the dissemination of ARGs through environmental systems. These models incorporate hydrological conditions, microbial community dynamics, and pollution loads to forecast how ARGs move through aquatic networks, serving as early warning systems for future outbreaks or ecological risks [85].

To support these analyses, automated pipelines like ARGs-OAP, ResFinder, AMRFinderPlus, and MetaStorm streamline the processes of ARG annotation, quantification, and visualization. These tools often include modules for quality control, taxonomy association, and user-friendly visualization dashboards, further reducing the technical barriers to comprehensive ARG surveillance [25].

**Figure 2:**
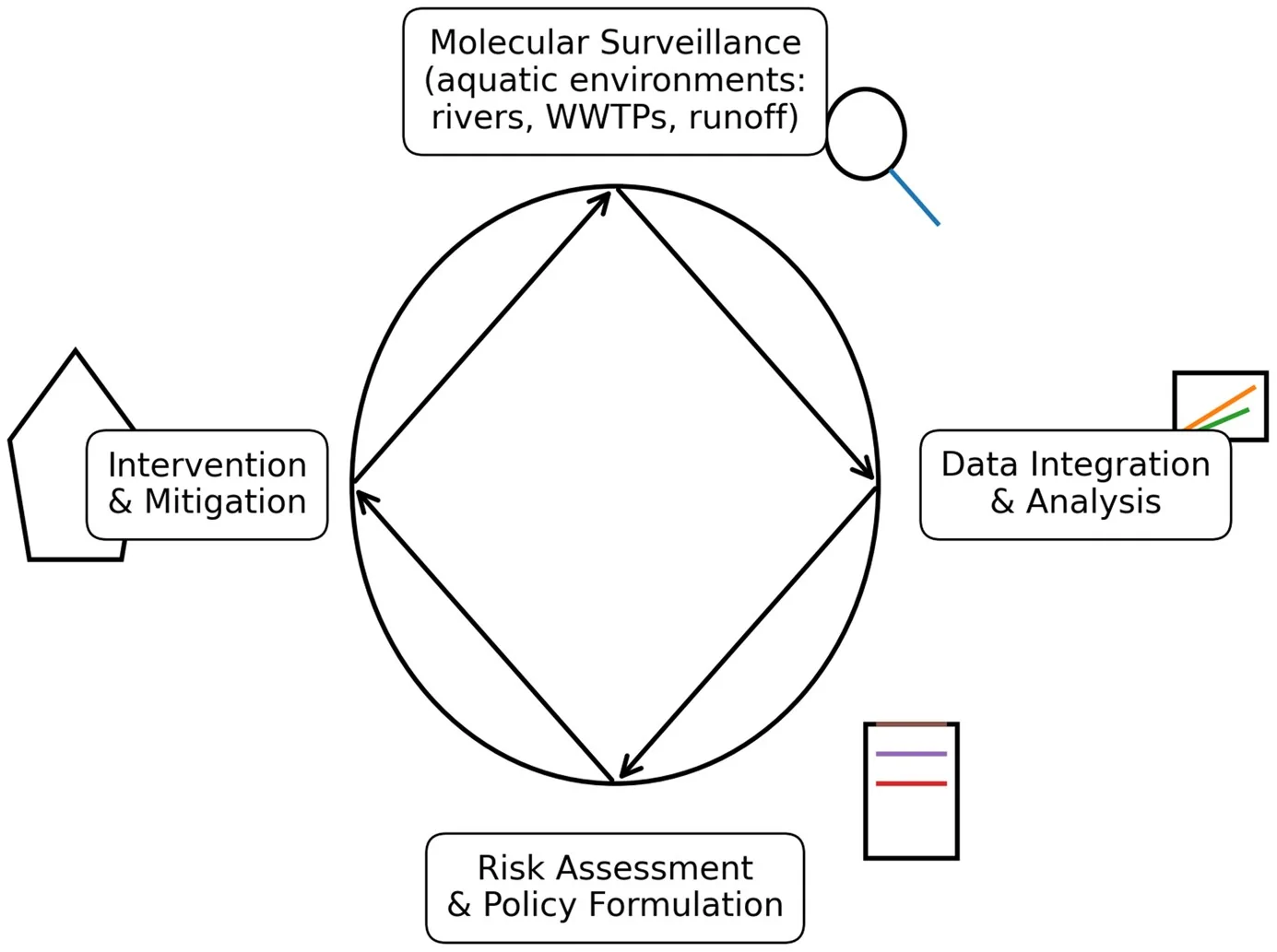
The One-Health approach cycle of aquatic AMR surveillance this circular framework illustrates the continuous and interconnected process of antimicrobial resistance (AMR) surveillance within aquatic environments, aligned with the One-Health approach.

While AI and ML offer immense potential, their effective application requires high-quality, well-curated training datasets and careful validation to avoid biases inherent in the training data. Nevertheless, their continuous development promises to significantly enhance our capacity for rapid, accurate, and comprehensive molecular surveillance of aquatic AMR, thereby strengthening One-Health initiatives [25] (Fig. 2).

This circular framework illustrates the continuous and interconnected process of antimicrobial resistance (AMR) surveillance within aquatic environments, aligned with the One-Health approach.

## Good practices for environmental AMR surveillance

As AMR emerges as a pressing global challenge, environmental monitoring, particularly in aquatic ecosystems, has become increasingly vital. Rivers, lakes, and wastewater systems act as reservoirs and dissemination pathways for ARGs, necessitating well-structured surveillance strategies. However, the effectiveness and comparability of resistome studies depend heavily on the adoption of standardized best practices. Without consistent protocols, data generated across different sites, time points, or research groups can be difficult to compare, limiting our ability to understand resistance trends, predict hotspots, or implement policy interventions [92].

A fundamental starting point for robust AMR surveillance is standardization of sampling methods (Table 3). This includes selecting representative sites, defining consistent sampling depths, volumes, and time intervals, and accounting for factors such as seasonality and hydrological conditions [86]. Resistome profiles can vary drastically between dry and wet seasons due to dilution effects and changes in runoff patterns [87]. Moreover, sampling from multiple environmental compartments such as surface water, sediment, and biofilms yields a more comprehensive picture, as ARGs may be differentially distributed across these matrices [88]. Equally critical is the implementation of rigorous metadata collection. Environmental parameters like pH, temperature, dissolved oxygen (DO), chemical oxygen demand (COD), heavy metal concentrations, and land use patterns must be recorded alongside microbiological data [89]. These contextual variables provide essential insights into the ecological drivers of resistance gene selection and dissemination, enabling researchers to draw meaningful correlations and build predictive models [90]. Longitudinal studies are another cornerstone of best practice. One-time sampling provides only a snapshot and may fail to capture temporal dynamics that influence ARG prevalence, such as rainfall, agricultural cycles, or industrial discharges [90]. By conducting repeated measurements over time, researchers can identify emerging trends, evaluate the impact of mitigation strategies, and establish baseline resistance levels for environmental risk assessment [87]. Another key principle is the integration of One-Health approaches, which recognize the interconnectedness of environmental, human, and animal health [91]. Environmental surveillance should not exist in isolation but rather be linked with clinical and veterinary AMR data [92]. Detection of carbapenem-resistant genes like blaNDM-1 in both rivers and hospital effluents suggests environmental contamination by healthcare sources and creates opportunities for cross-sectoral interventions [93]. Data sharing and transparency are also essential for maximizing the utility of AMR monitoring efforts [94]. Open-access data repositories such as ResistomeBank and NCBI’s Sequence Read Archive (SRA) or Pathogen Detection database allow researchers to deposit, curate, and retrieve resistome data across various ecosystems. These repositories support global AMR mapping, facilitate inter-study comparisons, and accelerate meta-analyses that can inform international policy [95]. Importantly, quality assurance measures must be integrated throughout the surveillance workflow. This includes the use of controls in DNA extraction and PCR processes, implementation of decontamination protocols to reduce false positives, and adherence to bioinformatics best practices for sequence filtering, assembly, and annotation [96]. Misannotations or contamination can lead to overestimation of resistance levels or misidentification of resistance mechanisms, which can misguide policy or risk assessments [97]. Additionally, effective AMR surveillance requires interdisciplinary collaboration. Environmental microbiologists, chemists, epidemiologists, data scientists, and policymakers must work together to design, execute, and interpret monitoring programs. Surveillance outcomes should be translated into actionable policy recommendations, such as the designation of high-risk zones, improvements in wastewater treatment infrastructure, or regulations on industrial discharges [98]. The success of environmental AMR surveillance depends on methodological rigour, cross-sector coordination, and commitment to transparency and standardization. By implementing these best practices, we can enhance the reliability of resistome data, better understand environmental contributions to the AMR crisis, and develop more effective strategies to safeguard public and ecological health [95].

**Table 3:** Recommendations for environmental AMR surveillance.

Phase	Recommendation	Key considerations	References
**Sampling design**	Standardize protocols	Representative sites; consistent depth, volume, timing; include seasonality and multiple matrices	[86, 88]
**Metadata collection**	Collect contextual data	Include physicochemical parameters (e.g. pH, DO, metals) and land use	[89]
**Study design**	Use longitudinal approaches	Capture temporal variability and establish baselines	[86]
**Quality assurance**	Apply strict QC measures	Include controls; ensure proper sequencing and bioinformatics workflows	[96, 97]
**One health integration**	Link cross-sector data	Integrate environmental, clinical, and veterinary AMR data	[91, 92]
**Data sharing**	Promote open access	Use public repositories for transparency and comparability	[95, 104]

## Policy and practical applications

The insights gathered from molecular surveillance of aquatic AMR are not merely academic; they hold profound implications for public health policy and practical applications within the broader One-Health framework [6]^.^ This paradigm recognizes that the health of humans, animals, and the environment is inextricably linked, necessitating integrated surveillance and intervention strategies [25]. Molecular tools provide the essential data to bridge these connections, allowing for a more holistic and evidence-based approach to AMR containment [99]. Environmental AMR surveillance, powered by molecular tools, plays a crucial role in providing early warning signals and identifying emerging threats that might later impact clinical settings [100]. By monitoring aquatic environments, which act as common mixing vessels for ARGs from various sources, policymakers can gain a comprehensive understanding of resistance circulation. Detection of carbapenem-resistant genes like blaNDM-1 in both rivers and hospital effluents directly suggests environmental contamination by healthcare sources and creates opportunities for cross-sectoral interventions [101].

Agricultural runoff represents another significant route for ARG dissemination. Investigations using qPCR and metagenomic techniques have documented the movement of resistance genes from livestock-treated soils into adjacent waterways, primarily due to the excessive or unregulated use of antibiotics in farming. These findings underscore the urgent need for policy reforms in agricultural antibiotic practices and the implementation of sustainable waste management systems to limit ARG transfer to aquatic ecosystems [102].

Moreover, molecular data is now being employed to identify geographical hotspots of resistance where ARG accumulation is notably high. This spatial mapping is crucial for conducting risk assessments, prioritizing intervention strategies, and allocating resources effectively [103]. However, to ensure the maximum utility of such surveillance, environmental data must be integrated into broader global monitoring initiatives. Programs like the World Health Organization’s Global Antimicrobial Resistance and Use Surveillance System (GLASS) are beginning to recognize the essential role of environmental surveillance in complementing clinical and veterinary datasets [104].

Open-access data repositories such as ResistomeBank and NCBI Pathogen detection serve as important platforms for storing, sharing, and curating resistome data from diverse ecosystems [95]. These repositories are complemented by reference databases like ARG-ANNOT, CARD, and ResFinder, which are used for annotating and identifying ARGs within the deposited data. Together, these resources not only facilitate inter-study comparisons and large-scale meta-analyses but also aid in tracking global trends in AMR, ultimately guiding international policy development [105].

To transform molecular surveillance into effective policy action, clear pathways must be established. This includes formally designating high-risk zones for intensive monitoring, upgrading wastewater treatment systems to remove ARGs and antibiotic-resistant bacteria (ARB), and enforcing stricter regulations on industrial and agricultural discharges that contain antibiotics, heavy metals, or other co-selective agents. Additionally, structured public health alert mechanisms are needed to communicate surveillance findings to both authorities and affected communities.

Ultimately, the successful translation of molecular surveillance data into impactful policy depends on interdisciplinary collaboration. Environmental microbiologists, chemists, epidemiologists, data scientists, and policymakers must work in concert to interpret complex data and craft evidence-based strategies. Such coordinated efforts are essential to mitigating the ecological and public health risks posed by antimicrobial resistance and to ensuring long-term resilience against its global spread [106].

## Ethical considerations in data sharing and pathogen detection

Beyond methodological and technical challenges, the increasing volume and sensitivity of molecular surveillance data for aquatic AMR raises significant ethical considerations, particularly concerning data sharing and the detection of potential pathogens [107]. While open-access platforms and global data sharing initiatives are crucial for comprehensive AMR mapping and meta-analyses, they also present challenges related to data privacy and responsible use. Environmental resistome data often contain information about the presence of clinically relevant ARGs and even specific pathogenic bacteria that could be linked to human health risks [108]. Ethical frameworks are needed to govern how such sensitive information is handled, especially when it might inadvertently reveal private information about communities or individuals, or when it could be misinterpreted by the public or non-experts.

Furthermore, the detection of specific pathogens or highly resistant strains in environmental samples raises questions about the responsibility of researchers and public health authorities to act on this information [1]. If a study identifies a highly virulent novel, multi-drug-resistant pathogen in a public water source, there is an ethical imperative to communicate this risk appropriately [109]. This involves managing the potential to cause public alarms without adequate context, ensuring data accuracy to avoid false positives, and establishing clear protocols for reporting and intervention [110]. Ethical guidelines must be developed to address the potential for misidentification, the implications of reporting findings that could impact public perception or economic activities like tourism and agriculture, and the need for a balanced approach that prioritizes public health without causing undue panic or stigmatization. These issues highlight the necessity for interdisciplinary discussions involving scientists, ethicists, policymakers, and local communities to establish clear best practices for responsible data management and communication in environmental AMR surveillance [109, 111].

## Conclusion

The integration of AI and omics-based technologies is reshaping the landscape of environmental AMR research by enabling deeper, more predictive insights into resistance gene dissemination and ecological drivers. Through advanced computational tools, including machine learning classifiers, source tracking algorithms, and metagenomic pipelines, researchers can now interrogate resistomes at unprecedented resolution. These innovations not only enhance our understanding of microbial dynamics and resistance gene mobility but also facilitate real-time surveillance, hotspot identification, and data-driven policy making. However, challenges remain in standardizing workflows, improving interpretability, and linking genotype to phenotype across environmental matrices. Addressing these gaps will require coordinated efforts in algorithm development, dataset curation, and cross-sector collaboration. Ultimately, AI-assisted multi-omics frameworks hold the promise to transform AMR risk assessment from a reactive to a predictive science, strengthening our ability to mitigate the global threat of AMR.
